# Pre‐Decisional Information Search in 2‐ to 4‐Year‐Olds: Young Children Select the Relevant Cues When Looking for a Hidden Reward

**DOI:** 10.1111/desc.70110

**Published:** 2025-12-11

**Authors:** Daniil Serko, Yi‐Lin Li, Nora Swaboda, Azzurra Ruggeri

**Affiliations:** ^1^ Technical University Munich Germany; ^2^ Central European University Vienna Austria; ^3^ University Potsdam Potsdam Germany

**Keywords:** active learning, decision making, cognitive development, cue selection, information search, pre‐decisional

## Abstract

This paper examines the capacity of 2‐ to 4‐year‐olds (*n* = 111, 56 female, age 25 to 59 months) to identify and select the most informative cue among several options when searching for a target—a task commonly used in decision‐making research and one in which young children have traditionally struggled. Across two experiments—including a preregistered replication—we tested children using a simplified version of Lindow's (2021) finding‐presents game. Children were presented with three cue cards (e.g., color, shape, icon) they could look up to determine which of the three boxes contained a hidden reward. Only one feature actually distinguished the target box—for example, the boxes all shared the same color, and all had a sticker on top, but differed in shape—so only one cue card offered genuinely informative guidance. In the first test phase, children were presented with the *same* set of boxes from the training phase. In the second test phase, children were presented with a *new* set of boxes and cue cards featuring a novel combination of cues. In Experiment 1, overall, children were more likely to select the relevant cue card, although 2‐year‐olds were successful only in the Same‐set test phase. Experiment 2 focused specifically on 2‐year‐olds and showed that they succeeded even in a version that ruled out the use of a cue‐saliency heuristic. Together, these findings reveal the early emergence of pre‐decisional information search abilities in preschoolers and introduce a straightforward, effective paradigm for assessing early information‐seeking skills.

## Summary

- Simplified design and visually engaging materials reveal young children's cognitive abilities in pre‐decisional information search often overlooked in traditional experiments.
- While 2‐year‐olds' success is inconsistent in Experiment 1, they successfully generalize cue selection to novel contexts in Experiment 2, revealing early sensitivity to informative cues.
- Three‐ and 4‐year‐olds successfully apply cue selection skills to both familiar and novel contexts, marking a clear developmental trajectory in information search efficiency.

## 1 Introduction

Before acting on information, we must first determine which kind of information will best serve our goals. Imagine a child on a mission to find the perfect chocolate cookie in a pantry full of jars that look suspiciously similar but contain different treats: A cookie jar with a red label reading “vanilla,” a cookie jar with a blue label reading “chocolate,” or a candy jar with a blue label reading “chocolate.” These jars differ along three key cues—jar type (cookie or candy), label text (vanilla or chocolate), and label color (red or blue)—each varying in how useful they are for identifying the desired snack.

To succeed, the child must prioritize cues strategically. While the blue label might initially draw attention (a salient but uninformative feature), the critical cues are the jar type (to exclude candies and focus on the desired cookies) and the text label (to identify which snack contains “chocolate”). In this example, only the combination of jar type and text is fully informative.

In simpler, more straightforward cases, only one cue matters. If the child were allergic to vanilla, the only cue that would truly matter in choosing a snack would be the “gluten‐free” sticker on the jar, regardless of its color or contents.

This ability to identify, search for, and select the most informative pieces of information—referred to as “cues” in decision‐making research—*before* making a decision is known as pre‐decisional information search (Lindow and Betsch 2018; Ruggeri and Katsikopoulos 2013; Svenson 1992). Identifying which cues are most relevant for reducing uncertainty is foundational to efficient and effective decision‐making, whether one is merely choosing a snack or evaluating the credibility of an Instagram post. This competence is especially critical for children, who must allocate their limited cognitive resources efficiently rather than wasting them on information that is not relevant or useful (Ruggeri and Katsikopoulos 2013).

Pre‐decisional information search refers to the strategic, goal‐directed process of gathering and selecting information directly relevant to making a specific decision (Golman and Loewenstein 2018; Lindow and Betsch 2018; Loewenstein 1994; Spektor and Wulff 2024). Gigerenzer and Todd (1999) describes this as a fundamental building block of decision making, because it constrains later choice by shaping the evidence set under consideration. Although related, pre‐decisional search is distinct from adjacent constructs such as exploration, hypothesis testing, and curiosity. Exploration denotes a broader process aimed at reducing uncertainty or expanding knowledge, which may aid decision making but often proceeds in a less structured or undirected fashion and is not necessarily anchored to an immediate choice goal (Hills et al. 2015; Meder et al. 2019, 2021). Hypothesis testing involves the systematic evaluation of conjectures, primarily oriented toward learning and theory building rather than resolving a particular decision; while the resulting knowledge may later inform choices, the process itself is conceptually distinct. Finally, curiosity represents an intrinsic motivational state that can trigger information acquisition or exploration, but it is not inherently tied to the pursuit of a specific decision.

Although numerous studies have examined pre‐decisional information search in preschoolers and older children (Bazhydai et al. 2020; Ghilardi et al. 2024; Herwig 1982; Mata et al. 2011; Ronfard et al. 2018; Ruggeri and Feufel 2015; Ruggeri and Lombrozo 2015; Török et al. 2024), comparatively little is known about the emergence of this ability in younger children (but see Lindow 2021; Lindow and Betsch 2018).

This is partly because most of the existing paradigms rely on advanced verbal or reasoning skills–such as question‐asking or abstract rule learning‐that often exceed the developmental capacities of toddlers and very young preschoolers (Betsch et al. 2018; Lindow and Betsch 2018).

This study addresses this limitation by examining whether children aged 2 to 4 years employ efficient pre‐decisional information‐search strategies when provided with child‐friendly study materials and simplified task designs that minimize verbal demands. By tailoring the task to better align with younger children's cognitive and verbal abilities, we aim to clarify the early developmental trajectory of this critical decision‐making competence.

### 1.1 The Early Emergence and Developmental Trajectory of Information‐Search Competences

Already from the first years of life, infants are selective about what kinds of information they attend to (for a review, see De Simone and Ruggeri 2022). Research suggests they follow a “Goldilocks” attentional pattern, allocating more attention to events of intermediate complexity while avoiding overly simple or complex stimuli (Kidd et al. 2012). When presented with events that violate infants‘ naïve theories of the world—such as gravity or object solidity—they spend more time attending and exploring objects that defy these expectations, like a toy car floating mid‐air or passing through a solid wall (Dunn and Bremner 2017; Stahl and Feigenson 2015). Importantly, by 12 months of age, infants are more likely to actively seek information from adults they perceive as knowledgeable when uncertain about a novel object. By selectively referencing the more knowledgeable partner in situations of epistemic uncertainty, infants demonstrate an early ability to identify credible and reliable sources of information, even before mastering verbal communication skills (Bazhydai et al. 2020).

Once children reach toddlerhood, they begin to respond to uncertainty and adapt their information‐search strategies to the information structure of the tasks they are presented with (Leckey et al. 2020; Poli et al. 2024; Török et al. 2024). For example, when faced with two confounded options— such as identifying a partially hidden object—toddlers often hesitate and gather more evidence rather than guessing randomly (Leckey et al. 2020). They shift their gaze back and forth between the two partially hidden images, spend more time looking at both options, and take longer to respond when the task is more difficult (i.e., when the images are more similar). Eye‐tracking data revealed that this increased deliberation and gaze switching reflected the toddlers’ efforts to accumulate more information before selecting an answer, rather than simply guessing or responding impulsively. Building on this, recent work by Poli et al. (2024) demonstrates that toddlers not only adapt their search strategies to the immediate demands of a task, but also flexibly adjust their exploratory behavior based on the underlying information structure. In a gaze‐controlled exploration task, children searched for a hidden animal in either a Uniform condition (where the animal could be in any of four locations) or a Skewed condition (where it was consistently in one location). By 24 months, toddlers showed more exploratory behavior than adults and tailored their information search to the task structure they faced: they engaged in broad visual searches in trials with high uncertainty (Uniform condition), but switched to simpler, more efficient searches when the solution was predictable (Skewed condition). These findings suggest that toddlers are not only sensitive to uncertainty, but can also flexibly deploy efficient search strategies that are well‐matched to the task at hand, supporting early learning and memory formation (see also Li et al. 2025).

During the preschool years, children's information search abilities become even more sophisticated. For example, Ruggeri et al. (2019) showed that 3‐ to 5‐year‐olds adapt their exploratory strategies based on the statistical structure of a task, selectively choosing between two actions—opening or shaking boxes—to find a hidden egg shaker. When the egg was equally likely to be in any of four boxes (Uniform condition), preschoolers tended to shake boxes first, engaging in broader exploration. Conversely, when the egg was most likely hidden in one specific box (Skewed condition), they employed more targeted strategies, being more likely to open the target box right away.

Preschoolers also show selectivity in whom they approach for information. For example, they prefer knowledgeable or confident social partners over uninformed ones (Harris et al. 2018; Mills 2013), and they differentiate between informants based on their competencies (Kushnir et al. 2013). In a study by Kushnir et al. (2013), 3‐ to 4‐year‐old children were presented with two agents: one who was good at fixing broken things, and one who was good at labeling new objects. By age 4, children selectively sought information from the fixer when presented with a broken toy, but turned to the labeler when encountering a novel object.

Building on evidence that preschoolers flexibly tailor their information search strategies to task demands (Ruggeri et al. 2019), recent work by Török, Domberg, et al. (2024) extends these findings by examining how children aged 3 to 7 adaptively select relevant cues in a problem‐solving context. In this study, children had to choose which of two monsters was better suited for a specific task (jumping hurdles vs. throwing balls) by uncovering either the monsters’ arms or legs—physical features predictive of their skills. The results showed that children's ability to selectively gather task‐relevant information improves with age, surpassing chance levels around 4 to 5 years old. This highlights the emerging capacity in early childhood not only to seek information but to strategically prioritize cues that are most informative given specific goals, further illustrating the developmental trajectory of adaptive and efficient information search.

### 1.2 A Contrasting View: Delayed Maturation of Efficient Pre‐Decisional Search

In contrast to the largely optimistic picture painted by recent developmental research—which highlights young children's emerging competence and adaptiveness in information search, especially when tested with age‐appropriate, child‐friendly paradigms—research on pre‐decisional information search coming from the decision‐making literature suggests that sensitivity to the informativeness of cues and truly efficient pre‐decisional information search may develop rather late, often reaching adult‐like levels only in late childhood or adolescence (Betsch et al. 2018; Davidson 1991, 1996; Mata et al. 2011). For example, Betsch et al. (2018) tested 5‐ to 10‐year‐old children and adults in a treasure‐hunt game, where participants selected which cues to consult to find a hidden treasure. Children were introduced to three animals, each differing in how reliably they predicted the treasure's location, but younger children struggled to integrate this probabilistic information into their search strategies, with performance approaching adult‐like efficiency only around age 9.

Similarly, Lindow and Betsch (2018) found that even 8‐ to 9‐year‐old children often failed to base their information search on relevant information from their environment. Children played a Piggy Bank Task, where they could open a limited number of cells on an information board to reveal the contents (i.e., coins) of three piggy banks before choosing one to open. In this task, even 8‐ to 9‐year‐olds frequently searched irrelevant information or overlooked key evidence, thus failing to consistently align their information searches with the most valuable option as efficiently as adults.

In line with these results, research indicates that 4‐ to 6‐year‐olds often struggle to judge when sufficient information has been gathered, frequently overestimating the informativeness of available evidence and overlooking the need for additional support (Fay and Klahr 1996; Klahr and Chen 2003). Children frequently ask unnecessary questions and make redundant choices, often continuing their search well beyond the point of certainty—even up to age 10—possibly, at least in some contexts, seeking confirming evidence to solidify their conclusions. This strategy, though inefficient, may reflect their role as novice learners navigating an uncertain environment (Chai et al. 2023; Ruggeri et al. 2016).

### 1.3 The Present Project

Our work addresses a critical gap in the literature by investigating whether 2‐ to 4‐year‐old children engage in efficient pre‐decisional information search, that is, whether they can strategically select which cues to consult before exploiting an information source. We directly address this by using a classic decision‐making paradigm from the literature: the treasure hunt game, where children must choose which clues to uncover in order to find a hidden reward.

Classic decision‐making tasks like the treasure hunt game differ in important ways from the tasks where young children have previously demonstrated early competence. Decision‐making paradigms typically require children to select among multiple cues that vary in informativeness, often involving abstract rules and probabilistic reasoning. These tasks demand integration of information across several steps and emphasize planning, risk evaluation, and strategic information gathering. In contrast, tasks that have shown success with young children tend to use visually concrete, engaging, and familiar scenarios closely tied to everyday experiences. Such tasks often allow for non‐verbal or intuitive responses, reducing demands on language or advanced reasoning, and provide clear, immediate feedback or opportunities for repeated exploration, which support learning and engagement.

Our approach preserves the core structure of a classic decision‐making task—the treasure hunt game— to directly assess children's pre‐decisional information search in a context comparable to prior research. In this simplified version, children searched for a reward hidden in one of three boxes. To disambiguate the reward's location, children were presented with three cue cards, only one of which identified the distinguishing feature of the target box. The other two cards were entirely uninformative, as they referred to features shared by all boxes. Thus, successful performance required recognizing and selecting the single informative cue. Children had up to two attempts. Performance across two attempts provides further insight into children's search processes: success on the first attempt signals robust pre‐decisional information‐search competence. Success only on the second attempt suggests a slower, less efficient—yet still meaningful—learning process, reflecting task understanding and sensitivity to minimal feedback, particularly in younger children. This design maintains the core challenge of selecting informative cues from among distractors, allowing us to assess children's ability to efficiently guide their information search.

Guided by the ecological active learning framework (Ruggeri 2022), we address key methodological limitations that may have underestimated young children's abilities in earlier studies. Specifically, we use child‐friendly instructions and age‐appropriate materials to ensure comprehension and motivation. We minimize unnecessary complexity and avoid requiring advanced verbal or mathematical skills, focusing instead on intuitive cue selection. Furthermore, we take into account children's own assumptions and strategies, recognizing that behaviors previously interpreted as inefficient may instead reflect adaptive approaches suited to their developmental stage and learning environment. By combining a classic decision‐making paradigm with an ecologically sensitive design, our project aims to provide a more accurate and fair assessment of whether and how young children engage in efficient pre‐decisional information search.

Specifically, we hypothesized that children would select the informative cue card significantly above chance, with performance improving with age. Success in the Same‐set test would indicate that children could apply the task logic after training, whereas success in the New‐set test would provide a stricter measure of generalization to a novel set of features.

## 2 Experiment 1

### 2.1 Methods

#### 2.1.1 Participants

Participants in Experiment 1 were 86 2‐ to 4‐year‐olds (*M* = 41.50 months, *SD* = 8.81 months; range: 25 to 59 months; 46 female) recruited in [blind for review] at a local museum, zoo, or through the participant database of the [blind for review] with testing conducted in the museum, zoo, or laboratory, respectively.

Note that, while ethnic and socio‐economic data were not collected for individual participants, the sample population in both studies was drawn from a community comprising approximately 71% ethnic German, 11% other European, 9% Middle Eastern, 3% Asian, 2% Afro‐German or Black African, and 4% other or unspecified backgrounds, representing a wide range of socio‐economic contexts.

An additional 28 children (15 female; *M* = 34.78 months; *SD* = 8.48 months; range: 24–59 months) were tested but excluded from the analyses because they were not able to focus on the task (*n* = 11), had language difficulties (*n* = 2), failed to pass the training phase (*n* = 1), experimenter error (*n* = 12), or parental intervention (*n* = 2). Most of these children were 2‐year‐olds (in total: 17 2‐year‐olds, 8 3‐year‐olds, 2 4‐year‐olds, one participant did not provide a date of birth).

Legal guardians gave written consent before participation. Children were asked for verbal assent before the study and received stickers for their participation. The study was approved by the ethics committee of the [blind for review].

The sample size was determined by conducting a priori power calculations via simulation for each planned statistical test. The most conservative estimate indicated a minimum overall sample of 80 children to detect a medium‐to‐large effect size (Cohen's *h* = 0.6) with 80% power using binomial logistic regression with a 0.05 criterion for statistical significance.

#### 2.1.2 Materials

**FIGURE 1:**
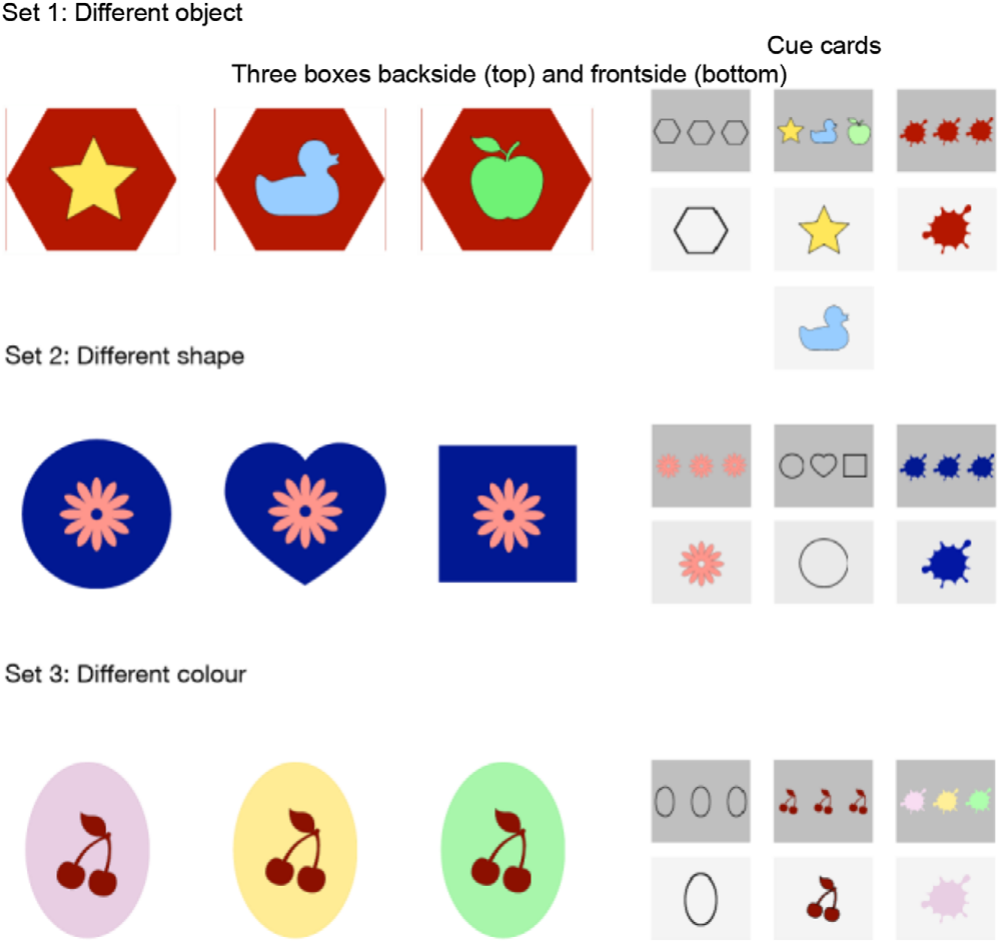
Picture showing all sets of boxes and the corresponding cue cards used in the experimental procedure. A sticker or a feather was placed in one box of each set as a reward.

All materials were custom‐designed and consisted of three sets of cardboard boxes, each paired with corresponding cue cards (see Figure 1 for pictures of all three sets). Each set consisted of three individual boxes with removable lids, characterized by a combination of color, shape, and icon printed on the lid. Within each set, the three boxes shared two identical features (e.g., color, icon) while differing in one specific feature (e.g., shape). Each set was defined by one distinguishing feature (see Figure 1). For example, in one set, all boxes were blue and displayed a flower icon on their lids but varied in shape (see Set 2 in Figure 1).

Each set of boxes was accompanied by a set of cue cards. The back of the card showed all possible variants of a specific feature, repeated as many times as they appeared among the boxes, while the front of the card depicted the variant corresponding to the target box (see Figure 1). For example, in Set 2, the *color card* showed three blue color splashes on its back (representing the three blue boxes) and a single blue splash on its front. The *icon card* displayed three flowers on its back (since all boxes featured a flower icon) and one flower on its front. The *shape card* depicted a square, a circle, and a heart on its back, while its front showed the critical shape needed to identify the target box (e.g., a circle if the target box was round‐shaped). One box in each set contained a feather or sticker as a reward.

#### 2.1.3 Design and Procedure

The experimental procedure included a training phase to familiarize children with the boxes and cue cards, followed by two test phases. Sets and target boxes were counterbalanced across participants.

##### 2.1.3.1 Training Phase

Children sat on a blanket and were presented with one set of boxes, which was placed on the blanket in front of them (see Materials section; see Figure 1). They were told that the goal of the game was to find out which box contained a small reward. The experimenter then introduced the children to the features of the boxes, highlighting the commonalities and differences among them. For the shared features, the experimenter pointed out that all boxes had the same color, shape, or icon. For the distinguishing feature, the experimenter explained how each box differed in that specific aspect, describing the unique characteristic of each box within the set. The order in which the differentiating feature was presented—either first or last—was counterbalanced across participants.

The experimenter shuffled the cue cards, introduced them as tools to identify the reward box, and placed one card after the other in front of the child, explaining each card provided information about a specific feature (e.g., color, shape, or icon). Once all the cards were laid out, the experimenter sequentially flipped them over, demonstrating how each revealed a feature of the target box. For each card, the experimenter highlighted the revealed feature, guided the child to identify all boxes possessing that feature, and clarified whether the feature was shared by all boxes or unique to one.

For example, referring to the distinguishing feature of Set 2 in Figure 1, the experimenter explicitly said, “Look, all the boxes have a different shape. This one is round, this one is heart‐shaped, this one is square. All boxes have a different shape.” When referring to the two remaining common features, the experimenter said, “But all boxes have the same color and all boxes have the same picture.” The experimenter then introduced the cue cards by saying: “You can't know where the surprise is hidden yet, can you? But I have a few cards with me. These cards will help us to find out in which box the surprise is hidden. This card tells us the color, this one the shape, and this one the picture of the box with the surprise. If we turn this card over, we know what color the box is. Aha, so the box with the toy inside is blue. If we turn this card over, we know what shape the box is. Aha, so the box with the surprise inside has a square shape. If we turn this card over, we know which picture is on the box. Aha, so the box with the toy has a flower on it! So now we know that the box with the surprise is blue, has a square shape, and has a flower on top. Do you know where the surprise is hidden?”

If the child was hesitant to respond, the experimenter provided prompts by pointing to the boxes and seeking confirmation for each one. After revealing all the features, the experimenter summarized the information to help the child deduce which box was the target.

All but one child (37 months old) completed this training phase by pointing to, or opening, the correct target box once the experimenter revealed the relevant cue card. During training, the experimenter offered corrective feedback when a child chose the wrong card or box, guiding them to the right answer.

##### 2.1.3.2 Same‐Set Test Phase

Children were presented with the *same* set of boxes from the training phase. The experimenter removed the cue cards and, while the child looked away, hid a new reward in one of the three boxes. The children were then told that a new reward was hidden, which could be in the same or a different box as before (the order of mentioning “same” and “different” was counterbalanced across participants). The experimenter reiterated that the reward's location was unknown but could be determined using the cue cards. After shuffling the cards, the experimenter placed them in front of the child and asked which feature—color, shape, or icon—they would like to know about, following the order of card placement.

Suppose in Set 2 all three boxes were blue and had a flower icon, but each had a different shape (square, circle, heart; see Figure 1). If the child selected a color cue card, the experimenter explained that since all boxes were blue, this would not help find the treasure. The cards were reshuffled, and the child could try again. If the child then picked the shape card, the experimenter revealed the feature on the back of it and asked whether they could select the target box. If instead the child selected another non‐informative card (e.g., the icon cue card), the trial ended without showing the reward. Thus, children had a maximum of two attempts per trial to select the distinguishing feature.

##### 2.1.3.3 New‐Set Test Phase

We introduced a new set of boxes, following the same procedure as the Same‐set test phase. The three new boxes could be distinguished by a different characteristic than before (e.g., by their shape if the target characteristic in the Same‐set phase was the icon on top of the boxes; see Set 1 and Set 2 in Figure 1). The test phase was conducted using the same steps and instructions as the first test.

This structure ensured that each trial required children to distinguish the single informative cue from uninformative alternatives, providing a direct test of their ability to identify relevant information. The exact script used during the procedure can be found in the Supporting Information.

### 2.2 Results

Children had three cue cards to choose from—only one of which was informative (see Figure 1). A random single choice would select the correct card 33% of the time. If children guessed twice—replacing the card after an incorrect first attempt—each guess would again have a 33% chance of being correct, resulting in an overall success rate of about 56%. Accordingly, 33% and 56% served as baseline chance levels in our binomial tests. Extending this logic to both tests, the expected probability of succeeding on both the Same‐set and the New‐set test is 30.9%. For the strict first‐attempt criterion, the chance of solving both tests on the first attempt is 11.1%.

In the Same‐set test, 52 out of 86 (60%, SE = 0.05) children selected the relevant cue card on their first attempt. An exact binomial test revealed that children's choices were significantly greater than chance (33%; *p <* 0.001; one‐tailed binomial test). A logistic regression analysis with age in months as a predictor for children's success at selecting the relevant cue card on their first attempt revealed no significant effect of age (*p* = 0.390, OR = 1.02 [0.97–1.08]).

Note that 18 additional children selected the relevant cue card on their second attempt. In particular, in the Same‐set test, five additional 2‐year‐olds selected the relevant cue card on their second attempt. An exact binomial test revealed that, if taking into account success on the first or second attempt, 20 out of 25 (80%) 2‐year‐olds’ choices were significantly greater than chance (56%; *p* = 0.011; one‐tailed).

**FIGURE 2:**
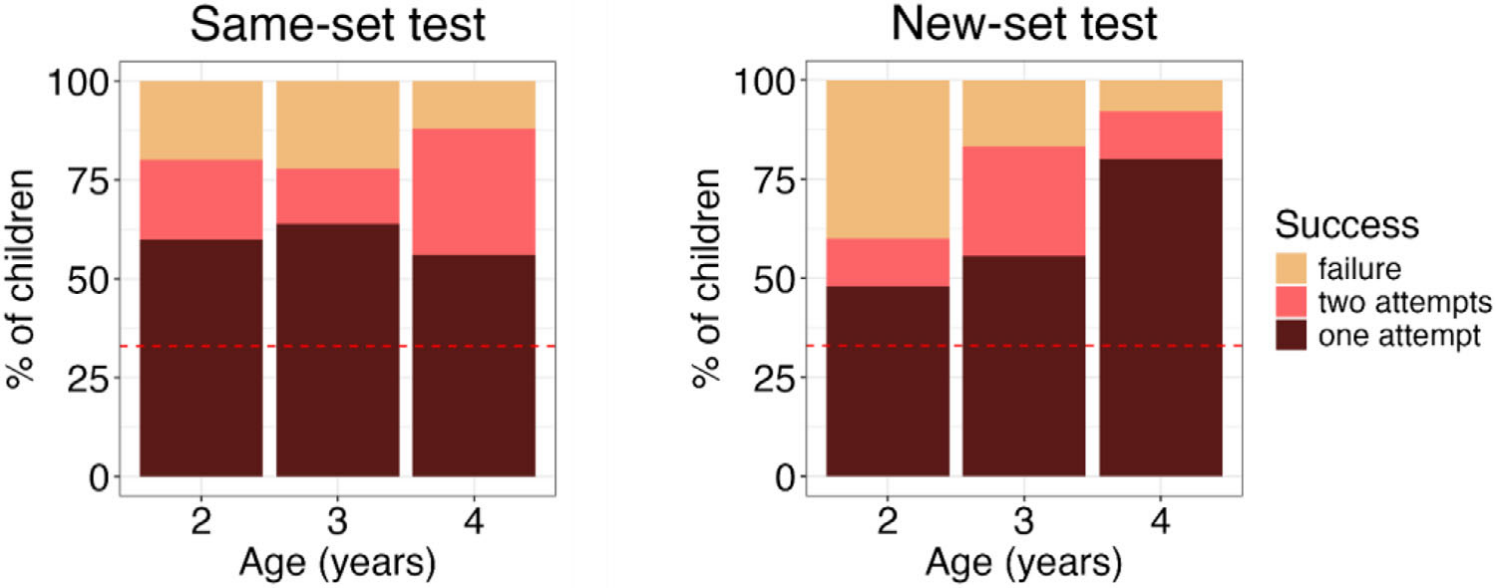
Proportion of children looking up the informative cue card by age group in the Same‐set test (left) and New‐set test (right). Colors indicate whether children looked up the informative cue card on the first or second attempt or not at all. The dashed red line indicates the chance level (33%).

In the New‐set test, 52 out of 86 (60%, SE = 0.05) children selected the relevant cue card on their first attempt. An exact binomial test revealed that children's choices were significantly greater than chance (33%; *p <* 0.001; one‐tailed). A logistic regression analysis with age in months as a predictor for children's success at selecting the relevant cue card on their first attempt revealed a significant effect of age (*p* = 0.003, OR = 1.09 [1.03–1.16]), indicating that older children were more likely to select the relevant cue card compared to younger children (see Figure 2).

In the Same‐set test, 15 out of the 25 2‐year‐olds (60%, SE = 0.01) selected the relevant cue card on their first attempt. An exact binomial test revealed that children's choices were significantly greater than chance (33%, *p* = 0.005, one‐tailed).

In the New‐set test, 12 out of the 25 2‐year‐olds (48%, SE = 0.01) selected the relevant cue card on their first attempt. An exact binomial test revealed that children's choices were not significantly greater than chance (33%; *p* = 0.086; one‐tailed).

In contrast, 20 out of the 36 3‐year‐olds (56%, SE = 0.08) and 20 out of the 25 4‐year‐olds (80%, SE = 0.08) selected the relevant cue card on their first attempt. An exact binomial test revealed that both 3‐ and 4‐year‐old children's choices were significantly greater than chance (33%; 3‐year‐olds: *p* = 0.004; 4‐year‐olds: *p <* 0.001; one‐tailed).

Note that 16 (19%) additional children selected the relevant cue card in the New‐set test on their second attempt. In particular, in the New‐set test, three additional 2‐year‐olds selected the relevant cue card on their second attempt. An exact binomial test revealed that, if taking into account success on the first or second attempt, 15 out of 25 (60%) 2‐year‐olds’ choices were not significantly greater than chance (56%; *p* = 0.423; one‐tailed).

Although Fisher's exact test showed no significant association between Same‐set and New‐set success at the first attempt (*p* = 0.825, OR = 1.11, 95% CI [0.42, 2.95]), the proportion of children who succeeded on both tests (Same‐set and New‐set) was far higher than expected under a chance baseline—54 of 86 children (62.8%) observed versus 30.9% expected when taking into account both attempts per test (*p* < 0.001, exact binomial test), and 32 of 86 children (37.2%) observed versus 11.1% expected for the strict, first‐attempt criterion (*p* < 0.001, exact binomial test)—demonstrating that performance was not random or inconsistent.

This pattern was stable across ages when applying the strict, first‐attempt criterion and comparing observed rates of success on both tests (Same‐set and New‐set) against the chance baseline for two independent first‐attempt guesses (1/9 = 11%; exact one‐sided binomial tests): 8 out of the 25 2‐year‐olds (32%), *p* = 0.004; 12 out of the 36 3‐year‐olds (33%), *p* < 0.001; 12 out of the 25 4‐year‐olds (48%), *p* < 0.001.

Moreover, age significantly predicted the probability of succeeding in both tests on the first attempt (*β* = 0.064, *p* = 0.019, OR = 1.07), showing that each additional month increased the odds of success by about 7%. Thus, even under the strictest first‐attempt criterion, success across the two test phases was significantly above chance at every age, with the strongest effects among 4‐year‐olds.

There was no effect of the particular set used on children's performance in any of the analyses (all *p >* 0.413).

### 2.3 Interim Summary of Experiment 1

**FIGURE 3:**
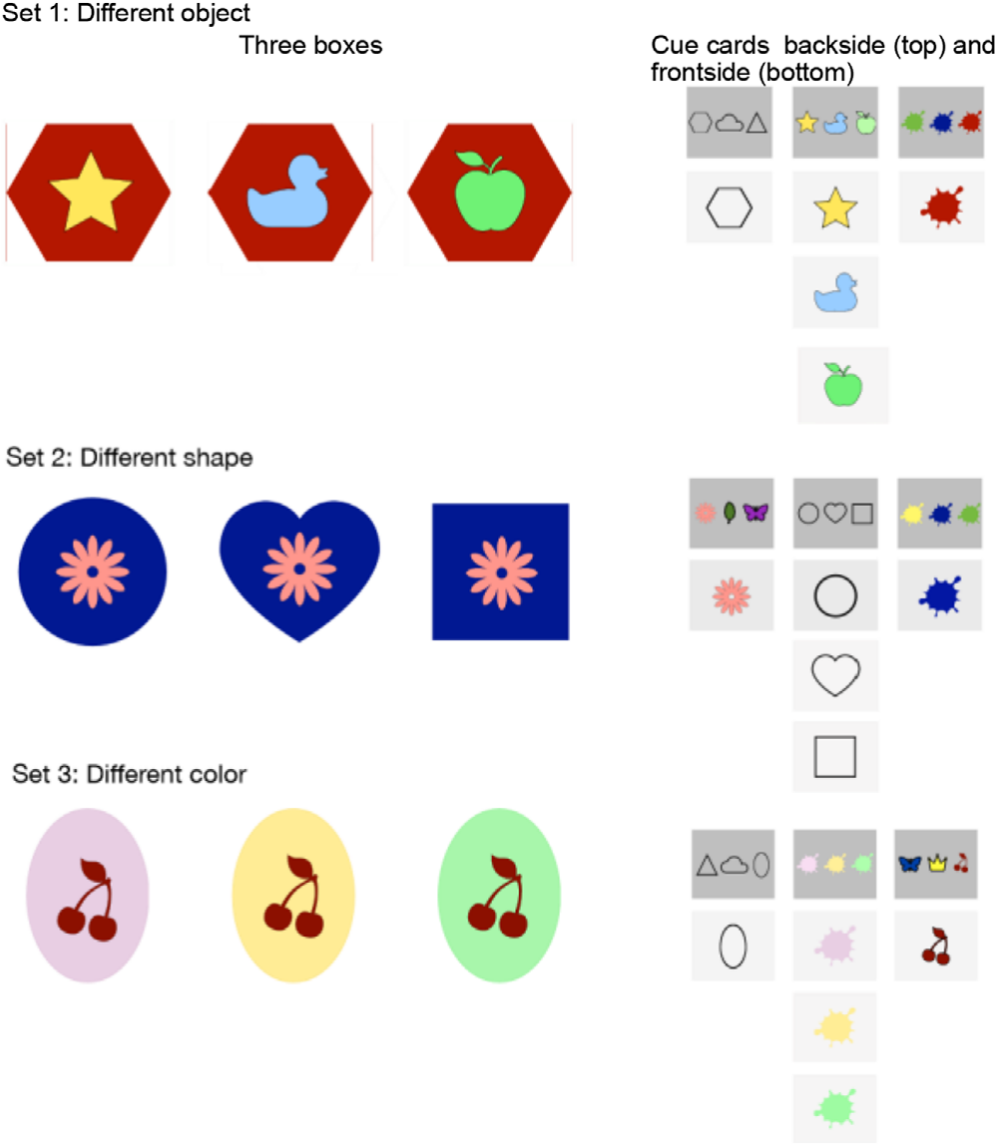
Picture showing all sets of boxes and the corresponding cue cards used in the experimental procedure of Experiment 2. A sticker or a feather was placed in one box of each set as a reward. Note that the pictures on the back of the uninformative cards differ as compared to the cards used in Experiment 1, where the pictures on the back of the uninformative cards were the same.

Our results demonstrate that, overall, 2‐ to 4‐year‐old children performed significantly above chance in selecting the relevant cue card. While age did not significantly predict performance in the Same‐set test, it emerged as a significant factor in the New‐set test. Specifically, 3‐ and 4‐year‐olds, but not 2‐year‐olds, were more likely than chance to select the relevant cue cards, suggesting a developmental change in the emergence of information search efficiency. Our findings suggested an alternative explanation: children may have relied on a simple heuristic rather than fully grasping the cards’ relevance. Specifically, they might have selected the cue card with varied symbols on its back (e.g., when all boxes shared the same color and icon but differed in shape, only the shape card featured different symbols), instead of meaningfully using the information (see Figure 1). While this approach would still reflect a sophisticated strategy for young children, we sought to investigate it further in Experiment 2. To do so, we modified the cue cards so that all cards, not just the relevant ones, displayed diverse symbols on their backs (see Figure 3). We focused on 2‐year‐olds for this follow‐up study, as they were the youngest participants in the initial experiment to approach above‐chance performance. In particular, on the Same‐set test, 2‐year‐olds performed significantly above chance if taking into account success on the first or second attempt (*p* = 0.011). By targeting this age group, we aimed to confirm that even the youngest children in our sample engage in rational pre‐decisional information search, rather than relying on simple visual heuristics.

## 3 Experiment 2

### 3.1 Methods

#### 3.1.1 Participants

Participants in Experiment 2 were 25 2‐year‐olds (*M* = 30.71 months; *SD* = 2.82; range: 25 to 35 months; 12 female) recruited and tested in [blind for review] a local museum.

An additional 18 children (*M* = 30.27 months; *SD* = 3.83 months; range: 24 to 35 months; eight female) were tested but excluded from the analysis because they were not able to focus on the task (*n* = 12), had language difficulties (*n* = 1), or experimenter error (*n* = 5).

Legal guardians gave written consent before participation. Children were asked for verbal assent before the study and received stickers for their participation. The study was approved by the ethics committee of the [blind for review]. The sample size matched that of the 2‐year‐old children in Experiment 1.

#### 3.1.2 Materials

Experiment 2 (OSF preregistration: https://osf.io/6h95e) retained the task structure and instructions from Experiment 1, but introduced a critical modification to the cue cards. In Experiment 1, two cards displayed identical symbols while one (the target card) featured different symbols (see Figure 1). For the follow‐up, we adjusted all cards to display three different symbols (see Figure 3), eliminating the potential confound of visual salience.

#### 3.1.3 Design and Procedure

The design and procedure of Experiment 2 were identical to those of Experiment 1.

### 3.2 Results

In the Same‐set test, 15 out of 25 (60%, SE = 0.10) children selected the relevant cue card on their first attempt. An exact binomial test revealed that children's choices were significantly greater than chance (33%; *p* = 0.005; one‐tailed binomial test).

A logistic regression analysis with age in months as a predictor for children's success at selecting the relevant cue card on their first attempt revealed no significant effect of age (*p* = 0.085, OR = 1.33 [0.98–1.93]).

In the New‐set test, 13 out of 25 (52%, SE = 0.10) children selected the relevant cue card on their first attempt. An exact binomial test revealed that children's choices were significantly greater than chance (33%; *p* = 0.038; one‐tailed binomial test).

A logistic regression analysis with age in months as a predictor for children's success at selecting the cue card on their first attempt in the New‐set test revealed no significant effect of age (*p* = 0.593, OR = 1.08 [0.81–1.46]). Note that eight additional children selected the relevant cue card on their second attempt.

Fisher's exact test showed no significant within‐child association (*p* = 1.000, OR = 1.14, 95% CI [0.17, 7.49]), but the observed rate of first‐attempt success in both tests (8 out of the 25 children, 32%) was significantly above the chance level of 11% (*p* = 0.004). These analyses demonstrate that group‐level reasoning was robust and well above chance, even though individual‐level stability could not be detected statistically due to limited power.

There was no effect of the particular set used on children's performance in any of the analyses (all *p >* 0.128).

## 4 Discussion

The ability to engage in pre‐decisional information search, that is, to distinguish between relevant and irrelevant sources of information is essential for any self‐guided and informed learner, regardless of age. Across two experiments, we find preliminary evidence that even 2‐year‐olds can engage in this kind of information search, although their performance was variable across tasks: they are sensitive to the informativeness of the information presented and actively select the most relevant cues to locate a hidden reward.

Experiment 1 showed that all age groups performed above chance in Same‐set tests, with 3‐ and 4‐year‐olds also exceeding chance in the New‐set test. This pattern suggests that while younger children were able to successfully engage in pre‐decisional information search in a familiar context, older children were more likely to generalize that knowledge efficiently to a novel context. Experiment 2 demonstrated that the performance observed in Experiment 1 was not due to a simple salience heuristic. By modifying the cue cards so that all cards—both informative and non‐informative—featured diverse symbols, we ruled out the possibility that success was driven by superficial visual cues. Even under these more stringent conditions, 2‐year‐olds performed significantly above chance. This provides strong evidence that their choices were guided by an understanding of the informativeness of the cue cards.

While our findings demonstrate that children as young as 2 years can select relevant cue cards, their success was not consistent across both experiments. Two‐year‐olds reliably passed the Same‐set test in both experiments, yet performed above chance on the New‐set test only in Experiment 2—even though the perceptual differences in Experiment 1 were arguably more salient. This puzzling pattern, with Experiment 1 performance hovering just below significance, calls for further work to identify the developmental and individual factors that drive such variation.

Although individual success in the Same‐set test did not reliably carry over to the New‐set test, the proportion of children who succeeded in both tests was robust. Even under the strictest scoring criterion—counting only first‐attempt responses—this joint success rate was significantly above chance across all age groups.

In contrast with previous work suggesting young children's information search is often inefficient (Betsch et al. 2018; Davidson 1991, 1996; Fay and Klahr 1996; Herwig 1982; Klahr and Chen 2003; Mata et al. 2011; Ruggeri and Feufel 2015; Ruggeri and Lombrozo 2015; Ruggeri et al. 2016), our results contribute to a growing body of evidence showcasing young children's emerging ability to search for information efficiently (Lindow 2021; Poli et al. 2024; Swaboda et al. 2022; Török et al. 2024).

For example, Lindow (2021) demonstrated that children as young as 5 could efficiently locate a hidden reward, with 80% focusing on informative cues and stopping their search after identifying relevant information. However, in stark contrast, our results reveal that with minor adjustments to stimuli, procedure and instructions, even much younger children can exhibit efficient search behavior, underscoring the critical role of task design in revealing these early competencies.

One possible reason we were able to reveal 2‐ to 4‐year‐olds’ pre‐decisional information‐search abilities is that our focus was on a specific subcomponent of information search—selecting relevant cues—rather than the entire, more complex search process. This narrower focus likely reduced cognitive load and made the task more manageable for 2‐year‐olds, whose ability to engage in pre‐decisional information search is not yet fully solidified, while still capturing the increasingly consistent performance observed among 3‐ and 4‐year‐olds.

Additionally, our methods were carefully tailored to their developmental level: we avoided the use of distractor cards and allowed children to respond either verbally or non‐verbally by pointing, minimizing both cognitive and verbal demands. Together, these design choices created an accessible and engaging task environment that better captured children's emerging capacities for efficient information search.

While our findings provide valuable insights into young children's pre‐decisional information‐search abilities, several limitations warrant consideration. First, children interacted with the same materials during both the practice and the Same‐set test phases, leaving open the question of how much prior exposure facilitated their successful engagement. Although children's strong performance in the New‐set test suggests some ability to transfer skills to novel contexts, future research should systematically investigate the amount and nature of practice required for young children to generalize these competencies.

Second, despite efforts in Experiment 2 to minimize visual salience and rule out superficial matching strategies, it remains possible that children relied on simpler heuristics rather than fully evaluating cue relevance. For instance, some irrelevant cue cards contained features absent from the test boxes (e.g., a triangle‐shaped box or butterfly sticker; see Figure 3), reflecting an unavoidable artifact of simplifying stimuli for very young participants. This limitation highlights the challenge of balancing ecological validity, task complexity, and developmental appropriateness in experimental design.

Third, it remains unclear at what age children begin to recognize when they have gathered sufficient information to make informed decisions. Prior research indicates that young children often continue searching beyond the point of certainty, engaging in unnecessary queries possibly due to underdeveloped uncertainty‐monitoring abilities or a preference for confirmatory testing (Ghetti et al. 2013; Lyons and Ghetti 2011; McCormack et al. 2016; Nickerson 1998; Ruggeri et al. 2016). While such behavior may appear inefficient, it could represent an adaptive strategy for novice learners navigating uncertain environments (Chai et al. 2023; Legare et al. 2013). Our current paradigm did not explicitly assess children's ability to monitor uncertainty or decide when to stop searching, leaving an important avenue for future exploration.

Fourth, we acknowledge that these exclusion criteria may reduce the generalization of our results, particularly among the youngest age group. It is possible that our criteria led to a final sample skewed toward children with relatively high cognitive and attentional capacities and stronger executive functioning. Testing exclusively in the lab could provide greater experimental control and likely reduce exclusion rates. However, restricting data collection to the laboratory would come at the cost of ecological validity and sample diversity. More broadly, future research should examine the extent to which our findings generalize not only to more heterogeneous child populations but also to different cultural and developmental contexts.

Building on these limitations, our findings suggest several promising directions for future research. Investigating how varying task complexity and cognitive load influence toddlers’ and young children's information search could help delineate the boundaries of their emerging capacities and identify key developmental milestones (Jones et al. 2021; Ruggeri et al. 2021; Swaboda et al. 2022). Additionally, examining individual differences in cognitive abilities, language skills, and other relevant factors may clarify why some children excel while others struggle with information‐seeking tasks. Longitudinal studies are particularly well suited to complement cross‐sectional findings by tracking how children refine their search strategies over time, adapt to more complex tasks, and how environmental influences such as formal schooling shape these developmental trajectories (Brod et al. 2017; Jones et al. 2021). For example, Brod et al. (2017) demonstrated that school entry significantly enhances executive functions, suggesting that similar environmental factors could impact the maturation of information‐search skills.

Moreover, developmental improvements in search efficiency appear linked to a decreasing tendency to make unnecessary queries, with preschoolers already showing some sensitivity to hypothesis space changes (Chai et al. 2023). Future work could extend our paradigm by allowing children, after revealing one cue, to choose whether to continue searching or make a decision. This manipulation would provide a valuable window into the early development of uncertainty monitoring and its influence on active learning strategies, while also elucidating the interplay between motivational factors and search efficiency.

Finally, future work should seek to conduct research that balances scientific rigor with age‐appropriate measures and interventions. One possible way to address this issue could be to reduce distractions at field testing sites and control for potential confounds such as differences in attention and executive functioning.

Our results compellingly demonstrate that children between 2 and 4 years of age are sensitive to the informativeness of available cues, with 2‐year‐olds showing emerging sensitivity and 3‐ and 4‐year‐olds already demonstrating more consistent and generalized use of relevance‐based reasoning. By systematically ruling out simpler explanations such as salience‐driven choices, we provide robust evidence that young children's information‐seeking behavior reflects an emerging, sophisticated understanding of cue relevance and quality. These findings not only challenge prevailing assumptions about early inefficiencies in children's information search but also highlight the critical role of carefully designed tasks in uncovering these early cognitive competencies. Ultimately, our work lays a foundation for future research and practical applications aimed at fostering efficient, self‐guided learning from the earliest stages of development.

## Funding

Daniil Serko was supported by a PhD scholarship from the Hans Böckler Foundation. Azzurra Ruggeri was supported by the grant “A Multifaceted Investigation of the Development of Intellectual Humility and its Links to Children's Learning Across Domains and Contexts” from the Templeton Foundation and by the grant “Towards a Science of Curiosity” awarded by the Volkswagenstiftung as part of the funding initiative “Artificial Intelligence and the Society of the Future.”

## Ethics Statement

All studies were approved by the Ethics Committee of the Max Planck Institute for Human Development, Berlin.

## Conflicts of Interest

The authors declare no conflict of interest.

## Supporting information




**Supporting File 1**: desc70110‐sup‐0001‐SupMat.pdf

## Data Availability

The data of this study are available on OSF (https://osf.io/6h95e).
