# Supplementary material for: Pre‐Decisional Information Search in 2‐ to 4‐Year‐Olds: Young Children Select the Relevant Cues When Looking for a Hidden Reward
Source: Dev Sci. 2025 Dec 11;29(1):e70110. doi: 10.1111/desc.70110 (PMC12699173; doi:10.1111/desc.70110)
Supplement: Supplementary file 1 — Supporting File 1: desc70110‐sup‐0001‐SupMat.pdf [file DESC-29-e70110-s001.pdf]

Script Treasure Hunt

Preparation

- Select training and test sets according to the list and prepare all three sets (draw target at random, hide surprise in correct box, prepare cards)
- Place training set on wooden beams - place boxes in the order of the different features on the map, symbols point to the child
- Clamp the envelope with the cards under the wooden beam, prepare the envelope with the other targets from the training set
- Sets for T2 and T3 out of sight, just like the stickers/springs

- - - - - - - - - - - -

1. **Training**

Hi, my name is xxx. Would you like to play a game with me?

Take a look at my three boxes here! I've hidden a surprise for you in one of the three boxes. In this game, we want to find out which box contains the surprise.

a. **Present boxes:** Order of common features randomized, target feature always first / last

Feature 1: (different feature)

Look, all the boxes have a **different picture** (a different color/different shape). This one is/has..., this one is/has..., this one is/has (short pauses, otherwise say it yourself)*. All boxes have a different picture on top (a different color / shape)!

* Possible variants S1: apple / duck / star - S2: a heart / round / square - S3: green / yellow / purple

Feature 2: (common feature)

But all boxes have the **same color** (the same picture/the same shape) (point to the boxes) They are/have all ..... (short pause so that children can answer themselves, otherwise say it yourself)* They all have **the same color**! (the same shape / the same picture on top)

Feature 3: (common feature)

And all boxes have the **same shape** (the same picture / the same color): They are/have all ..... (short pause so that children can answer themselves, otherwise say it yourself)* They all have the **same shape** (the same picture / the same color)!

* Possible variants S1: red / hexagon -- S2: blue / has a flower on it -- S3: egg / has cherries on it

[ALWAYS make sure that the child looks at the corresponding box for each of these, say very emphatically and, for example, trace contours or point to the picture].

b. **Present cards**

But you can't know where the surprise is hidden yet, can you?

If the child makes a strong assumption
But the boxes are closed and you can't even look inside. So you can only guess where the surprise is hidden. But you can't be sure!

But I have a few cards with me. These cards will help us to find out which box the surprise is hidden in. (Shuffle the cards and lay them down, saying:)

This card tells us the color, the shape and the picture of the box with the surprise (put the card down when speaking).

If we turn this card over, we know what **color** the box is. (turn over)

Aha, so the box with the toy inside is red.
Can you show me which boxes are red? (let child show

If child does not react/speak: Point to boxes and ask:
Is this box red / Is this box red / Is this box red?

These are all boxes, right?

If we turn this card over, we know what **shape** the box is. (turn over)

Aha, so the box with the surprise inside has six corners.
Can you show me which boxes have six corners / this shape? (Have the child show you, otherwise as above: Is this box ...

These are all boxes, right?

If we turn this card over, we know which **picture** is on the box. (turn over)

Aha, so the box with the toy has a star on it!
Can you show me all the boxes that have a star on them? (Have the child show you, otherwise as above: Is this box ...)

That's just this one, right?

So now we know that the box with the surprise is red, has this shape and has a star on top. (point to the three cards again - then pause for a moment)

Do you know where the surprise is hidden? (Let the child show you)

If the child does not react: Show the cards again, but if possible do not tell the child which is the correct box. - If children do not understand it even after repeated explanations, reveal it, continue and then eliminate it

Super! Would you like to take a look? (Place the box on a white surface, let the child open it)

You did a great job! Would you like me to hide another surprise for you? You can put your pen/sticker in the cup here, we'll keep the surprises there and you can take them home at the end.

2. **Test 1 (same set)**

Ok, then turn around/cover your eyes!

(Redraw target, hide surprise, prepare cards. Sometimes the same, sometimes a different target)

All right! I've hidden another surprise for you. It could be in the same or in a different (randomized) order. But you can't know where the surprise is hidden yet, can you?

To help you, I have these cards with me again. (Shuffle the cards and lay them down, saying:)

Would you like to know what color, shape or picture the box with the surprise has?

If the child doesn't say anything after a short time
Look, you can find out what color, what shape or what picture the box with the surprise has. What would you like to know?

Correct card revealed -> next set

So the box with the surprise has an apple on it.
Do you know where the surprise is hidden? (Let the child show you)

Correct box shown - > Hand over box, allow to open

Then take a look!
Well done! Do you fancy another round?

(Collect everything and get new boxes from the bag)

Wrong box shown - > second attempt

Hm, are you quite sure? There's an apple on the card, isn't there? Is there an apple on this box? (point to the box chosen by the child, possibly hold the card next to it for better comparison) Which box has an apple on it? (let the child show) Exactly, so the surprise is in this box, with the apple on it! Do you know where the surprise is hidden? (IMPORTANT: Have the child show you again - if wrong again, repeat from "Are you sure?" until the child chooses the correct box)

Wrong card revealed - > second attempt

So the box with the surprise is red. Hmmm... (pointing to each box individually) But that's all the boxes. That means we don't even know where the surprise is hidden yet. But you can try again.

(Pick up the cards and shuffle them again, then lay them down and say:)

Would you like to know what color, shape or picture the box with the surprise has?

Wrong card on the second attempt - > next set

So the box with the surprise is red. Hmmm... (pointing to each box individually) These are all the boxes. That means we don't even know where the surprise is hidden yet. But that's not so bad. I've got another set of boxes. We can try again with those.

(Collect everything and get new boxes from the bag, place them on the beams)

3. **Test 2 - new set**

Take a look. I've hidden another surprise for you in one of these boxes.

a. **Present boxes:** Order of common features randomized, <u>target feature always first / last</u>

<u>Feature 1: (different feature)</u>

Look, all the boxes have a **different picture** (a different color/different shape). This one is/has..., this one is/has..., this one is/has (short pauses, otherwise say it yourself)*. All boxes have a different picture on top (a different color / shape)!

* Possible variants S1: apple / duck / star - S2: a heart / round / square - S3: green / yellow / purple

<u>Feature 2: (common feature)</u>

But all boxes have the **same color** (the same picture/the same shape) (point to the boxes) They are/have all ..... (short pause so that children can answer themselves, otherwise say it themselves)* They all have **the same color**! (the same shape / the same picture on top)

<u>Feature 3: (common feature)</u>

And all boxes have the **same shape** (the same picture / the same color): They are/have all ..... (short pause so that children can answer themselves, otherwise say it yourself)* They all have the **same shape** (the same picture / the same color)!

* Possible variants S1: red / hexagon -- S2: blue / has a flower on it -- S3: egg / has cherries on it

[ALWAYS make sure that the child looks at the corresponding box for each of these, say very emphatically and, for example, trace contours or point to the picture].

b. **Test**

You can't even know where the surprise is hidden yet, can you?

If the child makes *a strong assumption*
But the boxes are closed and you can't even look inside. So you can only guess where the surprise is hidden. But you can't be sure!

To help you, I have these cards with me again. (Shuffle the cards and lay them down, saying:)

Would you like to know what color, shape or picture the box with the surprise has? (In the order in which the cards are lying there, starting with either R or L - as before)

If the child doesn't say anything after a short time
Look, you can find out what color, what shape or what picture the box with the surprise has. What would you like to know?

Correct card revealed -> next set

So the box with the surprise is purple.
Do you know where the surprise is hidden? (Let the child show you)

Correct box shown - > Hand over box, allow to open

Then take a look inside!
Well done! Do you fancy another round?

(Collect everything and get new boxes from the bag)

Wrong box shown - > second attempt

Hm, are you quite sure? The map shows purple, doesn't it?
Is this box purple? (point to the box chosen by the child, possibly hold the card next to it for better comparison)
Which box is purple? (let child show)
Exactly, so the surprise is in this box, which is purple!
Do you know where the surprise is hidden?
(IMPORTANT: Have the child show you again - if wrong again, repeat from "Are you sure?" until the child chooses the correct box)

Wrong card revealed - > second attempt

So, the box with the surprise has cherries in it. Hmmm... (pointing to each box individually) But these are all the boxes. That means we don't even know where the surprise is hidden yet. But you can try again.

(Pick up the cards and shuffle them again, then lay them down and say:)

Would you like to know what color, shape or picture the box with the surprise has?

At the end:

That was my game. Thank you for playing with me. I hope you enjoyed it!
